# Evidence from Cochrane systematic reviews for controlling the dissemination of COVID-19 infection. A narrative review

**DOI:** 10.1590/1516-3180.2020.029105062020

**Published:** 2020-07-06

**Authors:** Ronald Luiz Gomes Flumignan, Luis Carlos Uta Nakano, Patricia Irene Ferreira Pascoal, Brena Costa dos Santos, Rebeca Mangabeira Correia, Beatriz Périco Silveira, Fabio Akio Takihi, Carolina Dutra Queiroz Flumignan, Jorge Eduardo de Amorim, Álvaro Nagib Atallah

**Affiliations:** I MD, PhD. Vascular Surgeon and Adjunct Professor, Division of Vascular and Endovascular Surgery, Department of Surgery, Escola Paulista de Medicina (EPM), Universidade Federal de São Paulo (UNIFESP), São Paulo (SP), Brazil.; II MD, MSc, PhD. Vascular Surgeon and Adjunct Professor, Division of Vascular and Endovascular Surgery, Department of Surgery, Escola Paulista de Medicina (EPM), Universidade Federal de São Paulo (UNIFESP), São Paulo (SP), Brazil.; III MD. Vascular Surgeon and Volunteer Researcher, Division of Vascular and Endovascular Surgery, Department of Surgery, Escola Paulista de Medicina (EPM), Universidade Federal de São Paulo (UNIFESP), São Paulo (SP), Brazil.; IV MD. Resident, Division of Vascular and Endovascular Surgery, Department of Surgery, Escola Paulista de Medicina (EPM), Universidade Federal de São Paulo (UNIFESP), São Paulo (SP), Brazil.; V MD. Resident, Division of Vascular and Endovascular Surgery, Department of Surgery, Escola Paulista de Medicina (EPM), Universidade Federal de São Paulo (UNIFESP), São Paulo (SP), Brazil.; VI Undergraduate Medical Student, Escola Paulista de Medicina (EPM), Universidade Federal de São Paulo (UNIFESP), São Paulo (SP), Brazil.; VII Undergraduate Medical Student, Escola Paulista de Medicina (EPM), Universidade Federal de São Paulo (UNIFESP), São Paulo (SP), Brazil.; VIII MD, PhD. Vascular Surgeon and Volunteer Researcher, Division of Vascular and Endovascular Surgery, Department of Surgery, Escola Paulista de Medicina (EPM), Universidade Federal de São Paulo (UNIFESP), São Paulo (SP), Brazil.; IX MD, MSc, PhD. Vascular Surgeon and Adjunct Professor, Division of Vascular and Endovascular Surgery, Department of Surgery, Escola Paulista de Medicina (EPM), Universidade Federal de São Paulo (UNIFESP), São Paulo (SP), Brazil.; X MD, MSc, PhD. Physician and Full Professor, Division of Emergency Medicine and Evidence-Based Medicine, Department of Medicine, Escola Paulista de Medicina (EPM), Universidade Federal de São Paulo (UNIFESP), São Paulo (SP), Brazil; Founder and Director of the Cochrane Brazil Center, São Paulo (SP), Brazil.

**Keywords:** Coronavirus infections, Coronavirus, Review [publication type], Evidence-based practice, Pandemics, Quarantine, Personal protective equipment, Severe acute respiratory syndrome, Infection prevention protocols, Evidence synthesis, Rapid reviews

## Abstract

**BACKGROUND:**

COVID-19 infection has high transmissibility and several measures have been adopted for controlling its dissemination.

**OBJECTIVE:**

To identify and summarize the evidence from Cochrane systematic reviews (SRs) regarding measures for controlling the dissemination of COVID-19 infection.

**DESIGN AND SETTING:**

This review of Cochrane SRs was carried out in the Division of Vascular and Endovascular Surgery and in the Division of Emergency Medicine and Evidence-Based Medicine of Universidade Federal de São Paulo, Brazil.

**METHODS:**

A comprehensive search in the Cochrane Database of Systematic Reviews retrieved all Cochrane SRs directly related to measures for controlling COVID-19 dissemination. The main characteristics and results of all the SRs included were summarized and discussed.

**RESULTS:**

Three Cochrane SRs were included in the qualitative synthesis. These evaluated population-based and individual measures for controlling the dissemination of COVID-19.

**CONCLUSION:**

Low-certainty evidence shows that quarantine for people exposed to confirmed or suspected COVID-19 cases prevented 44% to 81% of incident cases and 31% to 63% of deaths, compared with situations of no measures. Moreover, the sooner the quarantine measures were implemented, the greater the cost savings were. High-confidence evidence showed that clear communication about infection control and prevention guidelines was vital for successful implementation. Low-certainty evidence showed that healthcare professionals with long gowns were less exposed to contamination than were those using coveralls. In addition, coveralls were more difficult to doff. Further SRs on controlling the dissemination of COVID-19 infection are desirable.

## INTRODUCTION

COVID-19 is a disease caused by a new type of coronavirus (SARS-CoV-2), which was identified in December 2019 in Wuhan province, China. Many patients develop only moderate symptoms without complications. However, approximately 14% of infected people develop a severe form of the disease that requires hospitalization and oxygen support and 5% of patients need treatment in intensive care units. SARS-CoV-2 disseminates among people mainly through the respiratory route, through coughing and sneezing, but it can also be transmitted by means of contaminated surfaces. Although the incubation time varies from 5 to 6 days in most cases, this time can reach up to 14 days. The infection period is not precise, varying from 24 hours to 48 hours before the manifestation of the symptoms. The viral load detected in the upper respiratory tract at the onset of the disease is high.^1^

When a new respiratory infection becomes widespread, as has occurred with the COVID-19 pandemic, healthcare professionals must adhere to prevention protocols in order to avoid contamination and infection. The prevention protocols recommend a variety of strategies: both personal measures, relating to the professionals, and measures that concern the environment. For the professionals, the protocols suggest that a mask, face shields, gloves and aprons, known as personal protective equipment (PPE), should be used. Concerning the environment, the protocols suggest that patients with respiratory diseases should be isolated and that cleaning routines should be more rigorous. Given that, in practice, adherence to these strategies can be challenging, healthcare authorities and facilitators need to support their professionals in implementing them.^2^

In epidemics and pandemics of highly infectious diseases such as Ebola, COVID-19 and severe acute respiratory syndrome (SARS), healthcare professionals’ contact with the body fluids of contaminated patients means that these professionals are the group at the highest risk of infection.^3^

The World Health Organization (WHO) recommends quarantine and isolation, in association with other public health measures, as measures for controlling the spread of infection. Both quarantine and social isolation are epidemiological interventions to mitigate infectious disease and reduce the potential for transmission. However, the effects of these and other measures for controlling the pandemic still generate discussion.^4^

Different countries have been using both individual and collective interventions for controlling the dissemination of infection, such as use of PPE, social isolation and compulsory quarantine, but the impact of these measures still needs to be studied in order to bring out robust evidence.

## OBJECTIVE

The aim of this study was to identify and summarize the evidence from Cochrane systematic reviews (SRs) regarding measures for controlling the dissemination of COVID-19 infection, in an overview.

## METHODS

### Design and setting

This review of Cochrane SRs was carried out in the Division of Vascular and Endovascular Surgery and the Division of Emergency Medicine and Evidence-Based Medicine of Universidade Federal de São Paulo, Brazil.

### Inclusion criteria

#### Types of study

We included full Cochrane SRs published in the Cochrane Database of Systematic Reviews (CDSR), with no restrictions on the date of publication. Withdrawn or outdated versions of SRs and protocols for SRs were considered not relevant.

#### Types of participants

All participants at risk of contagion, with suspected or confirmed clinical status of COVID-19 infection were considered relevant, i.e. males and females of all ages, with no restrictions as to the severity of the condition or place of treatment (outpatient or hospital setting).

#### Types of interventions

We considered SRs that evaluated any intervention to control the spread or reduce the contagion of COVID-19 infection, compared with standard care or another intervention, in at least one arm of the study.

#### Types of outcomes

Any epidemiological, clinical or laboratory results relevant to patients were considered, as assessed by the authors of the SRs included.

#### Search for reviews

We performed a sensitive systematic search in the CDSR, via Wiley, on April 26, 2020, using the MeSH terms ‘Coronavirus Infections’ and ‘Coronavirus’, and all related variants, along with free terms in ‘titles, abstracts and keywords’. The detailed electronic search strategy is shown in Table 1.

**Table 1.** Electronic search strategy and results in the Cochrane Database of Systematic Reviews

Line	Searched terms	Number of results
#1	MeSH descriptor: [Coronavirus Infections] explode all trees	38
#2	MeSH descriptor: [Coronavirus] explode all trees	11
#3	(severe acute respiratory syndrome coronavirus 2) or (Wuhan coronavirus) or (Wuhan seafood market pneumonia virus) or (COVID19 virus) or (COVID-19 virus) or (coronavirus disease 2019 virus) or (SARS-CoV-2) or (SARS2) or (2019 novel coronavirus)	68
#4	COVID-19 or (2019 novel coronavirus infection) or (COVID-19 pandemic) or (coronavirus disease-19) or (COVID19) or (2019 novel coronavirus disease) or (coronavirus disease 2019)	69
#5	Coronavirus* or Deltacoronavirus* or Deltacoronavirus* or (Munia coronavirus* HKU13) or (Coronavirus* HKU15) or (Coronavirus* Rabbit) or (Bulbul coronavirus* HKU11) or (Thrush coronavirus* HKU12)	154
#6	#1 or #2 or #3 or #4 or #5	172
#7	Filter: Cochrane Reviews	19

#### Selection of reviews

Two researchers (LCUN and RLGF) independently assessed the titles and abstracts to analyze whether the SRs met the inclusion criteria, using the Rayyan software (rayyan.qcri.org/welcome).^5^ Any disagreement was resolved in consultation with two other authors (CDQF and PIFP) or through discussion. Two authors (PIFP and RLGF) selected and summarized the SRs that were included.

#### Presentation of results

We presented the search results and the SRs that were included as a qualitative synthesis (descriptive approach).

## RESULTS

### Search results

Nineteen references were retrieved from our search strategy and, after screening the titles and abstracts, six SRs were preselected. After evaluating the full texts, three reviews were found to meet the inclusion criteria and were included in the qualitative summary.^2^^,^^3^^,^^4^

### Comments included

The most recent versions of all the SRs included were published in April 2020 in the CDSR. Details regarding the review design, characteristics of the interventions, comparisons, results and the certainty or confidence of evidence are presented in Table 2.^2^^,^^3^^,^^4^

**Table 2.** Details of review design, characteristics of interventions, comparisons, participants, main results and certainty of evidence, assessed by means of GRADE

Reference / Review design Types of primary studies analyzed in the review	Interventions	Comparisons	Participants	Main Results	GRADE
Nussbaumer-Streit et al.^4^/rapid review • Cohort • Case-control • Time series • Interrupted time series • Case series • Mathematical modelling studies	Different types and quarantine locations for individuals. They included studies combining isolation and quarantine.	• No quarantine. • Different types and quarantine locations. • Public health measures without quarantine to reduce the spread of the virus (isolation, social distancing, personal protective equipment, hand hygiene and others).	• (KQ1) contacts of a confirmed or suspected case of COVID-19 (SARS or MERS) or individuals living in areas with high rates of transmission; • (KQ2) individuals returning from countries with a declared outbreak of COVID-19 (SARS or MERS), defined by WHO as an ‘occurrence of cases of disease above normal expectations’.	• Quarantine of people exposed to confirmed or suspected cases prevented 44% to 81% of incident cases and 31% to 63% of deaths, compared with no measures (incident cases: four modelling studies on COVID-19 and SARS; mortality: two modelling studies on COVID-19 and SARS).	• Low certainty
				• The earlier the quarantine measures are implemented, the greater the cost savings (two modelling studies on SARS).	• Low certainty
				• The effect of quarantining travelers from a country with a reported outbreak was small with regard to reducing the incidence of illness and deaths (two modelling studies on SARS).	• Low certainty
				• When the models combined quarantine with other prevention and control measures, including school closures, travel restrictions and social distancing, modelling studies demonstrated a greater effect with regard to reducing new cases, transmissions and deaths than individual measures alone (incident cases: four modelling studies on COVID-19; subsequent transmission: two modelling studies on COVID-19; mortality: two modelling studies on COVID-19).	• Low certainty
Houghton et al.^2^/rapid review (synthesis of evidence) • Mixed method designs (qualitative aspect)	• Early recognition and source control (screening and breathing hygiene). • Administrative controls (isolation, spatial separation and cohort of patients). • Environmental and engineering controls (cleaning and disinfection; and ventilation). • PPE (dressing and undressing), aprons, gloves, masks and glasses). • Hand hygiene.	Control group is not evident from the nature of the review.	Most of the studies included involved nurses (14 studies) or doctors (9 studies). Other types of healthcare professionals included in the studies were occupational therapists, respiratory therapists and physical therapists; auxiliary personnel responsible for patient care, such as porters and domestic workers; laboratory technicians; infection control professionals; and managers.	• Healthcare professionals felt insecure about how to follow local guidelines when they were lengthy and ambiguous or did not reflect national or international guidelines.	• Moderate confidence
				• Clear communication about ICP guidelines was considered vital for its implementation.	• High confidence
				• Sufficient space to isolate patients was also considered essential for the implementation of the guidelines.	• Moderate confidence
				• The lack of PPE and poor quality equipment were serious concerns for healthcare workers and managers.	• Moderate confidence
				• Healthcare professionals believed that they followed ICP guidelines more closely when they saw their value.	• Moderate confidence
				• Healthcare professionals pointed out the importance of including all employees (cleaning, doormen, kitchen and other support staff) when implementing ICP guidelines.	• Low confidence
Verbeek et al.^3^/traditional systematic review • RCT • Non-randomized controlled trial • Cohort • Case-control • Prospective and retrospective controlled field studies	• Different types of full body protection (PPE), different compositions or amounts of PPE (body protection, such as aprons, overalls; eye and face protection in glasses, goggles, face mask visors or masks or hoods that cover the entire head; hand protection: gloves; and foot protection: boots). • Different parts of PPE or different procedures or protocols for placing and producing PPE. • Effectiveness of training to increase compliance with existing guidelines on the selection or use of PPE, including, but not limited to: education (courses); practical training; information only (such as posters, guidance leaflets, etc.); audit and feedback, or monetary or organizational incentives.	Comparisons were grouped according to similarity. Studies without a comparator group were not included.	• For simulation studies, any type of participant (volunteer or health professional) using PPE designed for Ebola virus disease or highly infectious diseases comparable with serious consequences was included. • For field studies, only studies carried out on healthcare professionals or auxiliaries exposed to patients’ body fluids in the form of splashes, droplets or aerosols contaminated with particles of highly infectious diseases that have serious health consequences, such as Ebola virus, SARS or COVID-19. Studies carried out on the laboratory team were excluded because the preventive measures in the laboratories are more detailed and easier to comply with.	• Using a respirator and energized air purifier with overalls can protect against the risk of contamination better than an N95 mask and gown (RR 0.27; 95% CI 0.17 to 0.43), but it was more difficult dressing (non-conformity: RR 7.5; 95% CI 1.81 to 31.1).	• Very low certainty
				• In an RCT (59 participants), people with a long gown had less contamination than those with a coverall, and the coverall was more difficult to wear	• Low certainty
				• The following modifications to the PPE design can lead to less contamination, compared with the standard PPE: combination of sealed gown and glove (RR 0.27; 95% CI 0.09 to 0.78), a more suitable fit around the neck, wrists and hands (RR 0.08; 95% CI 0.01 to 0.55), additional tags to grip, to facilitate the use of masks (RR 0.33; 95% CI 0.14 0.80) or gloves (RR 0.22; 95% CI 0.15 to 0.31).	• Very low certainty
				• better coverage of the wrist-cuff interface can lead to less contamination, compared with standard PPE (RR 0.45; 95% CI 0.26 to 0.78)	• Low certainty
				• Using the CDC recommendations can lead to less contamination, compared with no guidance (small spots: MD -5.44; 95% CI -7.43 to -3.45).	• Very low certainty
				• The use of additional computer simulation can lead to fewer errors in the process (MD -1.2; 95% CI -1.6 to -0.7).	• Very low certainty

GRADE: Grading of Recommendations, Assessment, Development and Evaluation; RCT = randomized controlled trial; PPE = personal protective equipment; KQ1 = key question 1; KQ2 = key question 2; SARS = severe acute respiratory syndrome; MERS = Middle East respiratory syndrome; WHO = World Health Organization; IPC = infection control and prevention; RR = relative risk; CI = confidence interval; CDC = Centers for Disease Control and Prevention; MD = mean difference.

### 1. Quarantine alone or in combination with other public health measures for controlling COVID-19: a rapid review ^4^

A rapid review was carried out to support WHO quarantine-related measures that were implemented subsequent to WHO’s declaration of the COVID-19 pandemic in March 2020. Nussbaumer-Streit et al. conducted a SR with abbreviated methods (rapid review) in order to evaluate two key questions (KQ): 1) the effects of quarantine (alone and in association with other public health measures) for individuals who had been in contact with confirmed cases of COVID-19; and 2) the effects of quarantine on individuals who had travelled from countries with a declared pandemic or who were living in regions with high transmission of the disease.

#### Main results

The authors of this SR included 29 studies as follows: 15 modelling studies on SARS and Middle East respiratory syndrome (MERS), four observational studies and 10 modelling studies on COVID-19 (Table 2). Because of the different measurement and analysis methods among the results of interest, it was not possible to carry out a meta-analysis, and the authors of this SR summarized the data in a narrative synthesis. Using the Grading of Recommendations, Assessment, Development and Evaluations (GRADE) approach, the certainty of evidence ranged from low to very low due to the type of evidence found through this SR.^6^

Simulated quarantine measures showed a benefit, as reported through the modelling studies. Quarantine measures for people exposed to confirmed or suspected cases prevented 44% to 81% of incident cases and 31% to 63% of deaths, compared with no measures. This low-certainty evidence was based on four modelling studies on COVID-19 in SARS (incident cases), and two modelling studies on COVID-19 in SARS (mortality). Two modelling studies on SARS suggested that the earlier the quarantine measures were implemented, the greater the cost savings would be (low-certainty evidence). Two modelling studies on SARS indicated that the effect of quarantine measures for travelers from a country with a reported outbreak was small, with regard to reducing deaths and the incidence of the disease (low-certainty evidence). Other prevention and control measures used in combination with quarantine, including travel restrictions, social distancing and school closures, showed a more significant effect in reducing deaths, transmissions and new cases, than did individual measures alone. Modelling studies on COVID-19 provided this low-certainty evidence: four studies relating to incident cases, two relating to subsequent transmission and another two relating to mortality. The studies on SARS and MERS were consistent with the results from studies on COVID-19.

#### Adverse effects

This SR focused on transmission, reduction of mortality and use of quarantine resources because WHO selected these as outcomes of interest. The authors of this SR did not include any consideration of the psychological impact of quarantine on individuals. There may be other adverse economic and health effects resulting from quarantine that were not assessed by this review (for example, domestic violence, unemployment and quality of life). For these reasons, this SR was unable to address the issue of when quarantine and other public health measures aimed at reducing the dissemination of COVID-19 should be relaxed or limited. It is also important to note that the authors of this SR did not subject the two modelling studies that reported on use of resources to specific critical economic assessments and did not attempt to come to any conclusions regarding the relative costs or efficiency of quarantine alone or in combination with other measures, compared with these public health interventions or measures in isolation.

#### Review conclusions

Although the evidence was limited to modelling studies, this SR showed that quarantine was essential for reduction of the incidence of COVID-19 and consequent mortality. Early implementation of quarantine and combination of quarantine with other public health measures seemed to be essential for ensuring effectiveness. Decision-makers should continuously keep the outbreak situation and the impact of the measures implemented under surveillance. To assess the true prevalence of infection and reduce the uncertainty of modelling assumptions, testing representative samples in different contexts might be of some help.

### 2. Barriers and facilitators to healthcare workers’ adherence with infection prevention and control (IPC) guidelines for respiratory infectious diseases: a rapid qualitative evidence synthesis^2^

This was a rapid review to synthetize the evidence relating to factors that influence healthcare professionals to follow infection control and prevention (ICP) protocols for respiratory diseases. These strategies include the use of PPE such as masks, face shields, gloves and aprons; isolation of patients with infectious respiratory disease; and stricter cleaning routines. The review authors searched only the MEDLINE database via OVID and included all types of primary studies, with no limits on date or language of publication. The review authors used the GRADE-CERQual approach (Trust in Evidence from Qualitative Research Reviews) to assess the level of confidence in each result.

#### Main results

The authors of this SR found 36 relevant studies, and 20 studies were included in the qualitative analysis of this review. There was no meta-analysis, and the results were reported narratively (Table 2). Two of the studies included were from Australia, four from America, four from Africa, and ten from Asia. The studies demonstrated the vision and experience of doctors, nurses and other health professionals who deal with SARS, influenza A (H1N1), MERS, tuberculosis (TB) or seasonal influenza. Most of the participating healthcare professionals were working in hospitals and primary care communities.

The following factors (barriers or facilitators) that were ascertained were based on results that were assessed as presenting a moderate to high level of confidence.

Lengthy and ambiguous guidelines or those that did not reflect national or international guidelines made the healthcare professionals insecure about how to follow local guidelines. Continual changes to the local guidelines left the healthcare professionals feeling overwhelmed. They also described how ICP strategies led to increased workloads and fatigue; for instance, because they had to wear PPE and do additional cleaning. The level of support that the healthcare professionals felt that they received from their management team was described as a point that influenced their responses to ICP guidelines.

Clear communication about ICP guidelines was considered vital. The healthcare professionals pointed out that there was a lack of training on how to use PPE and on the infection itself. They also considered it to be a problem when training was not mandatory.

Sufficient space to isolate patients was also considered essential. The lack of isolation rooms, antechambers and showers was a problem. Other critical practical measures described by the healthcare professionals included rapid screening of infected patients, minimization of overcrowding, easy access to handwashing facilities and restrictions on visitors.

The healthcare workers and managers pointed out that the lack of PPE and inadequate quality equipment were serious concerns, and that there was a need to adjust the volume of supplies as outbreaks of infection continued.

The healthcare professionals believed that they followed ICP guidelines more carefully when they understood their value. Some of the healthcare professionals felt motivated to follow the guidelines because they felt responsible for their patients or because they were afraid of infecting themselves or their families. The healthcare workers identified that there was some trouble in using masks and related PPE when it made patients feel isolated, scared or stigmatized. The healthcare professionals also found that the masks and other equipment were unpleasant to use. The culture of the workplace also possibly influenced whether the healthcare professionals followed ICP guidelines or not.

In many of the conclusions, the healthcare professionals pointed out the importance of including all employees, including cleaning, doorkeeping, kitchen and other support staff, when implementing ICP guidelines.

#### Adverse effects

Some factors possibly constitutes barriers against infection control and prevention strategies, such as lack of alignment between national and international protocols, which led to insecurity among health professionals with regard to following these protocols. Another critical factor was the lack of personal protective equipment or the availability only of inferior material, which caused discomfort among professionals. In some situations, although healthcare professionals were aware of the regulations, it could be challenging to adhere to the protocols, especially when working under critical conditions.

#### Review conclusions

The review authors pointed out several factors that influenced the ability and willingness of healthcare professionals to follow ICP guidelines when managing respiratory diseases. Those factors included points associated with the guidelines themselves and how they were disclosed, support from managers, training, workplace culture, physical space, access to and confidence in PPE and the intention to provide excellent patient care. The review also highlighted the importance of including all staff at the facility, including the support team, when implementing the ICP guidelines.

### 3. Personal protective equipment for preventing highly infectious diseases due to exposure to contaminated body fluids in healthcare staff^3^

In situations of outbreaks of epidemics or pandemics, there is an increased risk of infection for healthcare professionals due to greater exposure to body fluids from infected patients. Use of PPE can reduce this exposure. This study was a SR that assessed which type of full-body PPE and which method of dressing and undressing presented the lowest risk of contamination and infection for healthcare professionals. The study also addressed training methods that had the capacity to increase adherence to protocols.

#### Main results

The authors of this SR included 24 studies with 2,278 participants: 14 of these studies were randomized controlled trials (RCTs), nine had a non-randomized design and one was a quasi-RCT. Among the 24 studies included, eight compared dressing and undressing processes, eight compared types of PPE, three evaluated types of training and six evaluated adapted PPE. In 18 simulation studies on harmless microbes or fluorescent markers, the average contamination rates were 25% for the intervention group and 67% for the control group.

The certainty of the evidence for all the results was very low, except when mentioned otherwise, because it was based on only a few studies, because it used indirect evidence (simulation) and because of the risk of bias in the studies included (Table 2).

Use of an energized respirator and air purifier with coveralls can better protect against the risk of contamination than a N95 mask and gown (relative risk (RR) 0.27; 95% confidence interval (CI) 0.17 to 0.43), but it was more difficult to wear (non-conformity: RR 7.5; 95% CI 1.81 to 31.1). In an RCT (59 participants), long aprons (gowns) were better able to protect against contamination than overalls, and the overalls were more difficult to wear (evidence of low certainty). Long aprons (gowns) were also better able to protect against contamination than short aprons (small spots: mean difference (MD) -10.28; 95% CI -14.77 to -5.79). PPE made of more breathable material led to a similar number of stains on the trunk (MD 1.60; 95% CI -0.15 to 3.35), compared with water-repellent material, but user satisfaction was possibly higher (MD -0.46; 95% CI -0.84 to -0.08, scale from 1 to 5).

The following modifications to the design of the PPE had the capacity to lead to less contamination, compared with the standard PPE: 1) a more suitable fit around the neck, wrists and hands (RR 0.08; 95% CI 0.01 to 0.55); 2) a combination of sealed gown and gloves (RR 0.27; 95% CI 0.09 to 0.78); 3) better coverage of the wrist interface (RR 0.45; 95% CI 0.26 to 0.78; low-certainty evidence); and 4) additional tags to grab to facilitate the use of masks (RR 0.33; 95% CI 0.14 0.80) or gloves (RR 0.22; 95% CI 0.15 to 0.31).

Use of the recommendations of the Centers for Disease Control and Prevention (CDC) had the capacity to lead to less contamination, compared with no guidance (small spots: MD -5.44; 95% CI -7.43 to -3.45). One-step removal of gloves and gown did not enable lower contamination with fluorescence (RR 0.98; 95% CI 0.75 to 1.28) than separate removal. However, this one-step removal strategy led to less bacterial contamination (RR 0.20; 95% CI 0.05 to 0.77). Use of double gloves had the capacity to lead to less viral or bacterial contamination, compared with simple gloves (RR 0.34; 95% CI 0.17 to 0.66), but not less contamination with fluorescence (RR 0.98; CI 95 % 0.75 to 1.28). Additional spoken instructions had the capacity to lead to fewer errors in execution (MD -0.9; 95% CI -1.4 to -0.4) and fewer points of contamination (MD -5; 95% CI -8.08 to - 1.92).

Computer simulation in addition to other measures had the capacity to lead to fewer errors in the process (MD -1.2; 95% CI -1.6 to -0.7). A video lecture on PPE placement had the capacity to lead to better skill scores (MD 30.70; 95% CI 20.14 to 41.26) than a traditional lecture. Face-to-face instructions had the capacity to reduce non-compliance with guidelines, compared with just providing folders or videos (odds ratio (OR) 0.45; 95% CI 0.21 to 0.98).

#### Adverse effects

The use of various elements of PPE created discomfort regarding its use, which gave rise to possibly higher risk of contamination of the healthcare professional at the time of undressing.

Use of an energized respirator and air purifier with overalls was more challenging to wear (non-compliance: RR 7.5; 95% CI 1.81 to 31.1).

#### Review conclusions

This SR found very low to low-certainty evidence that covering more parts of the body enabled better protection, but that protection covering more of the body was generally more difficult to wear or make and was associated with less user comfort and, therefore, would perhaps even lead to more contamination. More breathable types of PPE could lead to similar contamination but could also lead to greater user satisfaction. Modifications to the design of the PPE, such as incorporation of gripping tags, had the capacity to decrease the risk of contamination. Placement and manufacturing procedures that followed the CDC guidelines, the ability to remove the gloves and gown in one step, use of double gloves, use of verbal instructions during execution and disinfection of gloves had the capacity to reduce contamination and increase compliance. Face-to-face training in the use of PPE had the capacity to reduce errors more than training based on printed material such as folders.

The authors of this SR concluded that there was a lack of RCTs with long-term follow-up; a lack of simulation studies with large numbers of participants to find out which PPE combinations and which procedures gave better protection; and a lack of evidence from real life. They considered that a consensus regarding simulation of exposure and evaluation of the results was necessary. Therefore, they considered that the use of PPE by healthcare professionals who would be exposed to highly infectious diseases needed to be recorded, and that these professionals should be followed up prospectively regarding the risk of infection.

## DISCUSSION

The COVID-19 pandemic is right now the most significant global health threat. Its dissemination has been rapid, with at least 146 countries affected.^7^

One of the WHO guidelines for disease control is quarantine, which means separation of healthy people who might be infected by the virus and have the potential to spread the disease. Other similar recommendations are isolation (similar to quarantine, but including people with symptoms of COVID-19) and social distancing (when healthy people keep a physical distance away from other people).^1^

In massive pandemics with a highly infectious disease such as COVID-19, there is higher contamination among healthcare professionals. These individuals may develop infectious conditions earlier, due to their more significant contact with infected people. Therefore, it is a matter of urgency to determine strategies and develop protocols for these professionals so that there is greater adherence to safety regulations. When PPE such as masks, glasses, face shields, gloves, aprons and coveralls is routinely used within the care provided for these infected patients and the guidelines for dressing and undressing are followed, cases of contamination will be more significantly mitigated. However, these strategies often become difficult to follow in practice, and so there is a need for more significant support for these professionals, for them to be implemented.

Several measures have been taken in the light of this pandemic. These have included combinations of case isolation, domestic quarantine and social distancing among at-risk groups (the elderly and individuals with comorbidities). These are the most effective combined policies for reducing the epidemic curve.

Through these Cochrane SRs, it was possible to identify the effects of the strategies that have been used to clarify how PPE should be best used among healthcare professionals and how valuable this is. When the care protocols for avoiding contamination were used correctly, these professionals were safer. Also, it was possible to determine the effects of quarantine (alone and in association with other public health measures) for reducing the incidence of COVID-19 and the resultant mortality. Early implementation of quarantine was found to be essential for the effectiveness of this action.

Nonetheless, the success of these approaches comes not only from the effectiveness of their implementation, but also especially from the natural and biological history of the COVID-19 pathogen, its transmissibility and the feasibility of interventions within the context of the country’s public health organizations.^8^

The number of Cochrane systematic reviews that directly address the COVID-19 pandemic is still limited, but efforts are being made to rapidly produce high-quality syntheses of evidence that are of interest for decision-makers within healthcare and healthcare policies.^9^

## CONCLUSION

After a comprehensive systematic search, three Cochrane SRs were included in this review. They contributed evidence regarding population-based measures (such as quarantine and isolation) and individual measures (such as use of PPE, type of PPE, etc.) for controlling the spread of COVID-19.

Low-certainty evidence showed that quarantine for people who had been exposed to suspected or confirmed cases prevented 44% to 81% of incident cases and 31% to 63% of deaths, compared with no measures. Moreover, the earlier that the quarantine measures were implemented, the higher the cost savings were.

Evidence with high confidence showed that clear communication about ICP guidelines was considered vital for their implementation. In addition, evidence of moderate confidence showed that healthcare professionals felt insecure about how to follow local guidelines when these did not reflect national or international guidelines or when they were lengthy and ambiguous. Sufficient space to isolate patients was also seen as essential for the implementation of the guidelines.

Low-certainty evidence showed that people with long aprons received less contamination than those with coveralls, and that the coveralls were more challenging to wear. Furthermore, there was low-certainty evidence that better coverage of the wrist cuff interface had the capacity to lead to less contamination, compared with standard PPE. Uncertainty remained regarding the best use of PPE for controlling COVID-19 dissemination because the evidence relating to this was of very low certainty.
